# Seven Day Insertion Rest in Whole Body Vibration Improves Multi-Level Bone Quality in Tail Suspension Rats

**DOI:** 10.1371/journal.pone.0092312

**Published:** 2014-03-17

**Authors:** Rui Zhang, He Gong, Dong Zhu, Jiazi Gao, Juan Fang, Yubo Fan

**Affiliations:** 1 Department of Engineering Mechanics, Jilin University, Changchun, Jilin, People’s Republic of China; 2 Department of Orthopedic Surgery, No. 1 Hospital of Jilin University, Changchun, Jilin, People’s Republic of China; 3 School of Biological Science and Medical Engineering, Beihang University, Beijing, People’s Republic of China; Faculdade de Medicina Dentária, Universidade do Porto, Portugal

## Abstract

**Objective:**

This study aimed to investigate the effects of low-magnitude, high-frequency vibration with rest days on bone quality at multiple levels.

**Methods:**

Forty-nine three-month-old male Wistar rats were randomly divided into seven groups, namely, vibrational loading for *X* day followed by *X* day rest (VL*X*R, *X = *1, 3, 5, 7), vibrational loading every day (VLNR), tail suspension (SPD), and baseline control (BCL). One week after tail suspension, rats were loaded by vibrational loading (35 Hz, 0.25 g, 15 min/day) except SPD and BCL. Fluorescence markers were used in all rats. Eight weeks later, femora were harvested to investigate macromechanical properties, and micro-computed tomography scanning and fluorescence test were used to evaluate microarchitecture and bone growth rate. Atomic force microscopy analyses and nanoindentation test were used to analyze the nanostructure and mechanical properties of bone material, respectively. Inductively coupled plasma optical emission spectroscopy was used for quantitative chemical analyses.

**Results:**

Microarchitecture, mineral apposition rate and bone formation rate and macromechanical properties were improved in VL7R. Grain size and roughness were significantly different among all groups. No statistical difference was found for the mechanical properties of the bone material, and the chemical composition of all groups was almost similar.

**Conclusions:**

Low-magnitude, high-frequency vibration with rest days altered bone microarchitecture and macro-biomechanical properties, and VL7R was more efficacious in improving bone loss caused by mechanical disuse, which provided theoretical basis and explored the mechanisms of vibration for improving bone quality in clinics.

## Introduction

Bone tissue is a complex composite biological material with the ability for functional adaptation. Mechanical environment is an important factor in controlling and influencing bone structure. Osteoporosis is a systemic skeletal disease characterized by low bone mass and microarchitecture deterioration of bone tissue, with a consequent increase in bone fragility and susceptibility to fracture [1]. Many factors contribute to onset of bone loss. In addition to hormone deficiency, microgravity can also lead to bone loss. For astronauts staying four to six months in space, the mineral content of lower limb bones decreases remarkably, and the rate of loss of bone mineral density (BMD) is almost 1.6% per month [2], [3]. In a gravitational environment for the duration of space travel, deterioration of some bone structures is irreversible though the bone mass has started to increase [4].

Pharmacologic treatment is the most popular intervention to prevent osteoporosis, but is unsuitable for all patients because of potential side effects [5]. Thus, non-invasive and non-pharmacologic therapy is a focus of current research in osteoporosis treatment [6], [7]. Low-level mechanical stimuli can improve both quantity and quality of trabecular bone in 6 to 8 years old female sheep, which (if applicable in humans) may serve as an effective intervention for osteoporosis [8]. After daily mechanical stimulation of the hindlimbs of adult sheep for a year with 20 min bursts of very-low-magnitude, high-frequency vibration, the density of trabecular bone in the proximal femur significantly increases (by 34.2%) than those in controls [6]. A pilot randomized controlled trial in disabled children demonstrated that low-magnitude, high-frequency mechanical stimuli are anabolic to trabecular bone, and may provide a non-pharmacologic treatment for bone fragility [9]. In addition, clinical studies of bone responses to exercise, bed rest, and microgravity confirmed the sensitivity of bone to physical and environmental stimuli [10]. The beneficial effects on bone mass and strength can be attributed to the sensitivity of bone cells to mechanical stimuli. However, bone cells lose mechanical sensitivity soon after stimulation, and a rest interval between each low-magnitude load cycle can create a potent anabolic stimulus [11]–[13]. Compared with daily loading, Ma et al. found that low-magnitude, high-frequency mechanical vibration is more effective in improving bone microarchitecture and biomechanical properties in ovariectomised rodents if the long-duration mechanical stimulus is separated by several rest days [14]. However, the influence of vibration with rest days on osteoporosis caused by mechanical disuse remains unknown. Long-term limb disuse may disorder normal bone metabolism. Bone tissue is below or next to the threshold of bone remodeling, and the amount of bone resorption in remodeling process is greater than bone formation, which results in decreased bone mass. However, this is different from osteoporosis caused by the decline of ovarian function in women after menopause, which may increase the mechanical set point of mechanostat [15]. Because of the different nature between disuse osteoporosis and estrogen-deficient osteoporosis, the bone qualities in response to this new type of low-magnitude, high-frequency intermittent mechanical intervention strategy proposed by Ma et al. [14] need to be further investigated through mechanical disuse model for the osteoporosis caused by weightlessness.

The mechanical properties of bone are determined by not only the structure and geometry, but also the tissue properties of bone material itself [16]. Considerable evidence demonstrated that bone mass and microarchitecture are sensitive to mechanical stimuli, and a feedback regulatory mechanism between external load and metabolism must exist [17]. This mechanism needs to be explored thoroughly using multi-level investigations, i.e. mechanical testing or micro-computed tomography (micro-CT) for bone qualities (e.g., elastic modulus or BMD) [18]. Atomic force microscopy (AFM) was recently used to improve our knowledge of the nanostructure of bone material because the dimensions of many microarchitecture features of interest in bone tissue are several micrometers or less [19]–[25]. In addition, elastic properties of bone microarchitecture components differ from the macroscopic values, and nanoindentation is a mechanical microprobe method that allows the direct simultaneous measurement of elastic modulus and hardness of the material [26]–[29].

The previous studies on the effects of low-magnitude, high-frequency vibration on bone quality mainly concerned one or several aspects of bone property (e.g., mechanical property, mineral content, or microarchitecture) [6]–[10], [14], [30], but little is known about macro-micro-nano multi-level bone quality in response to the low-magnitude, high-frequency vibration with rest days, which was essential for better understanding the underlying mechanism for this mechanical intervention strategy. Accordingly, this study aimed to explore the effects of low-magnitude, high-frequency mechanical vibration on bone quality at multiple levels when the long-duration mechanical stimulus was separated by several rest days rather than daily loading. Macro- and micromechanical and morphological investigations were performed in this study to determine the bone mechanical properties, nanostructure of bone material, and material properties of femora with mechanical methods separated by 1, 3, 5, and 7 d rests in the loading cycle compared with daily loading in tail suspension rodents.

## Materials and Methods

### Materials

This study was performed in strict accordance with the recommendations of the Laboratory Animal Standardization Committee. The protocol was approved by the Medical Ethics Committee of No. 1 Hospital of Jilin University (2013-145). All efforts were made to minimize suffering of animals.

**Figure 1:**
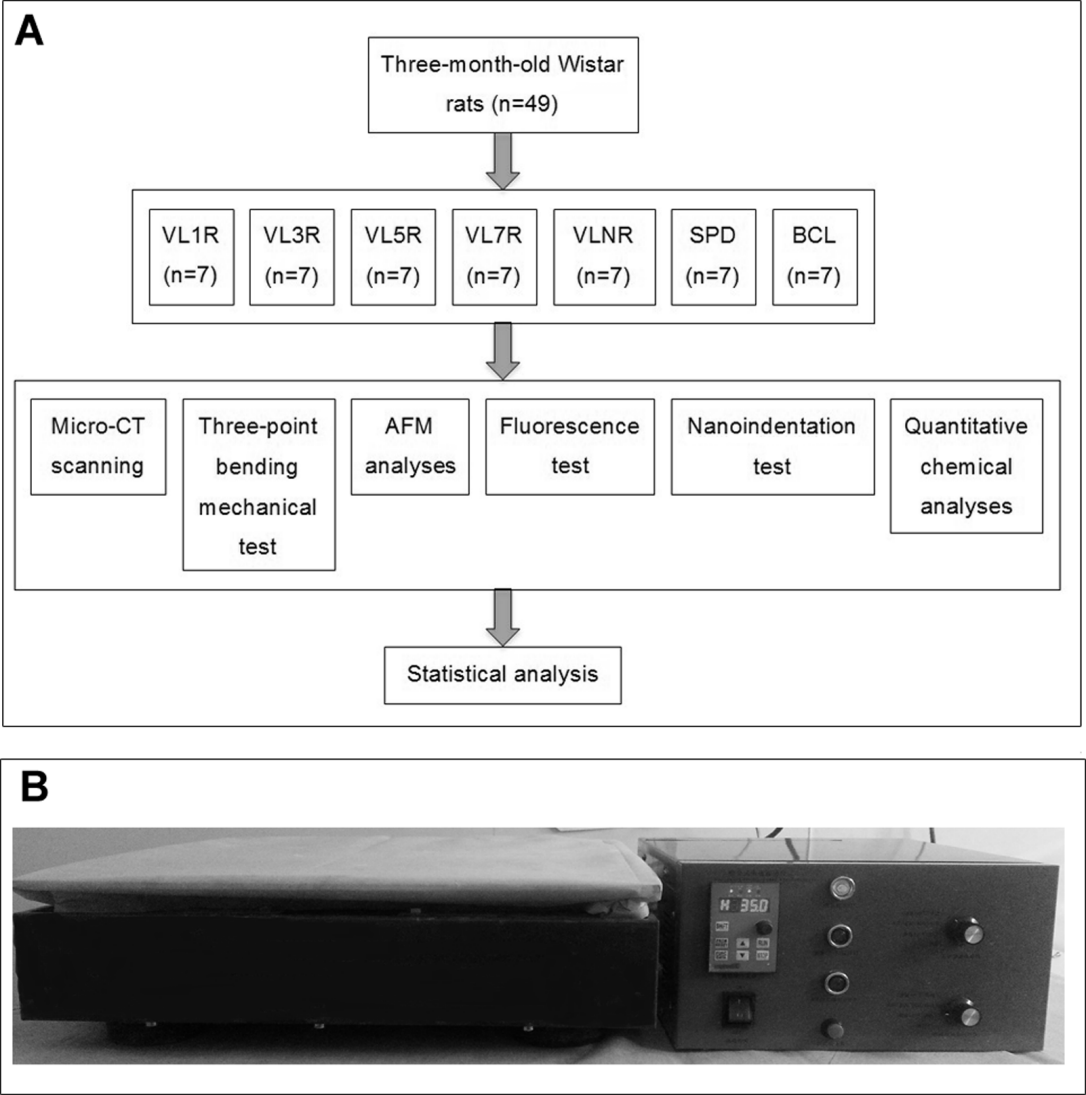
Temporal schematic of experiment and equipment for vibrational loading. (A) The temporal schematic; (B) The equipment for vibrational loading, which was assembled manually with a vibrational platform and a controller.

A total of 49 three-month-old male Wistar rats were purchased from the Experimental Animal Center of Jilin University. These rats were housed as singletons, and provided with a standard rodent diet (autoclaved NIH-31 with 6% fat; 18% protein; Ca:P, 1∶1; and fortified with vitamins and minerals) and tap water during the experimental period. The environmental temperature was 24±2°C in natural light condition. All rats were randomly divided into seven groups, namely, vibrational loading for *X* day followed by *X* day rest (VL*X*R, *X = *1, 3, 5, 7), vibrational loading every day (VLNR), tail suspension (SPD), and baseline control (BCL). The temporal schematic was shown in Fig. 1A. Non-invasive tail suspension was applied on all rats except those used for baseline control (BCL, n = 7), which were fed without any treatment. One week after tail suspension, high-frequency, low-magnitude whole body vibration was performed for eight weeks, which was similar with the study of Ma et al. [14]. The animals that were loaded by whole body vibration stimuli (35 Hz, 0.25 g, 15 min/day) on the first day were given one rest day, that is, vibrational loading with 1 d rest (VL1R, n = 7). Further groups were similarly created as follows: vibrational loading for 3 d followed by 3 d rest (VL3R, n = 7), vibrational loading for 5 d followed by 5 d rest (VL5R, n = 7), vibrational loading for 7 d followed by 7 d rest (VL7R, n = 7), and no rest day or vibrational loading every day (VLNR, n = 7). The rats in the tail suspension group (SPD, n = 7) were suspended without mechanical loading during the eight-week experimental period. The equipment for vibrational loading was assembled manually with a vibrational platform and a controller whose frequency and acceleration were adjustable (Fig. 1B). The weights of rats were measured before the experiment, one week after the suspension, and per week during the experiment. At day 42 and day 43 of the experimental protocol, all rats were treated with subcutaneous injection of calcein (dose: weight, 5 mg:1 kg) and tetracycline (dose: weight, 30 mg:1 kg), respectively. At day 52 and day 53 of the experimental protocol, calcein and tetracycline were re-injected to create a fluorescence maker [31], [32]. All the rats were sacrificed at eight weeks, and the femurs were prepared for tests following removal of skin, muscle, and tendons.

### Micro-CT Scanning

Left femurs were initially fixed with 80% ethanol (EtOH). Then, quantitative analysis of microarchitecture of trabecular bone in the femoral head was performed with micro-CT scanning (Skyscan 1076, Skyscan, Belgium). The spatial resolution for specimen scanning was set to 18 μm. The microarchitecture parameters of trabecular bone in the femoral head, such as bone volume fraction (BV/TV), trabecular thickness (Tb.Th), trabecular number (Tb.N), trabecular separation (Tb.Sp), and BMD were calculated by CTAn (CTAn, Skyscan, Belgium) [33].

### Three-point Bending Mechanical Test

After micro-CT scanning, left femurs were cleaned in normal saline and a three-point bending mechanical test was performed on each left femur using an electronic universal testing machine (AG-X plus, Shimadzu, Kyoto, Japan). The test was performed with a fulcrum span of 20 mm and an actuator speed of 1 mm/min. Failure load was recorded, and energy absorption was determined as the area under the force-deflection curve until the point of failure. The elastic modulus was calculated using the following equation:
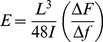
(1)where *L* is the fulcrum span, 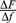 is the slope of force-deflection curve, and *I* is the moment of inertia of an area. The cortical thickness of the fracture position was measured for calculating the elastic modulus.

### AFM Analyses

After three-point bending mechanical test, sections of the left proximal femurs were cleaned in normal saline and dehydrated in increasing EtOH concentration from 85% to 100%. Longitudinal cortical bone specimens with a thickness of 2 mm were cut along the femoral shaft axis from femur shafts with a low-speed diamond saw under constant deionized water irrigation. Longitudinal trabecular bone specimens with a thickness of 1 mm to 2 mm were similarly cut from femur heads along the femoral neck axis.

Each bone sample (after 5 min of washing in ultrasonic bath, and natural drying) was placed horizontally onto the sample disk and imaged by a Multimode quadrex SPM with a Nanoscope IIIe controller (Veeco Instruments, USA). Imaging was operated under ambient conditions in standard AFM tapping mode using a commercial Silicon AFM probe (Tap300AI-G, BudgetSensors Instruments, Bulgaria) with a 125 μm cantilever length, a 40 Nm^–1^ constant force, a 300 kHz resonant frequency, and a tip radius lower than 10 nm. The size of mineral grains, i.e. the collagen bundles and the hydroxyapatite crystals, was measured using NanoScope Analysis version 1.4.0, as described by Milovanovic et al. [25].

### Fluorescence Test

Right femurs were initially fixed with 80% EtOH, and dehydrated similar to left proximal femurs. Then, right femurs were embedded separately in polymethylmethacrylate (PMMA), ensuring that all bones were not demineralized. Each embedded right femur was sectioned into proximal femur and femur shaft. The right proximal femurs were cut along the coronal plane with a low-speed diamond saw under constant deionized water irrigation, which exposed the cancellous bone. Right femur shafts were then cut along the horizontal plane using a similar method, which exposed the cortical bone. Slices for fluorescence test were cut from right femur shafts. Then, the remaining parts were used for nanoindentation test. The mineral apposition rate (MAR) and bone formation rate (BFR/Tb.Ar) were calculated under fluorescence microscopy [34]. Laser scanning confocal microscopy (FV500, Olympus Corporation, Japan) was used to take pictures of analyzed fields, and one picture per right femur was obtained, and a total of 49 fluorescence images were obtained. MAR and BFR/Tb.Ar were measured by Image-Pro Plus software.

### Nanoindentation Test

In this study, elastic modulus (*E*) and hardness (*H*) of longitudinal and transverse trabecular bone material, as well as those of longitudinal cortical bone material, were measured. Longitudinal trabecular bones with a thickness of 2 mm cut from left femoral heads were used for nanoindentation test in the longitudinal direction, and were also embedded separately in PMMA. Longitudinal cortical bones with a thickness of 2 mm cut from right femur shafts were used for nanoindentation test in the longitudinal direction, and transverse cancellous bones with a thickness of 2 mm of right femur heads were tested in the transverse direction. All the embedded samples were metallographically polished using silicon carbide abrasive papers of decreasing grit size (600, 800, 1500, and 2000 grit), and finally on microcloths with finer grades of diamond suspensions to the finest, 0.05 μm grit, to produce smooth surfaces for nanoindentation test. Specimens were washed in deionized water between each polishing step to remove debris. Nanoindentation tests were performed using Nano Indenter G200 (Agilent Technologies, Ltd., Santa Clara, CA, USA). A sharp Berkovich diamond indenter, a three-sided pyramid with the angle of 76°54′ between two edges, was used for all measurements. The specimens to be examined were located in the microscope and positioned beneath the indenter using the x–y table. The indenter was then slowly driven toward the surface at a constant displacement rate of 10 nm/s until surface contact was detected by the changes in the load and displacement signals. After contact, a permanent hardness impression was made by driving the indenter into the specimen to a depth of 1000 nm at a constant loading rate of 750 μN/s, holding at this load for a period of 10 s and then unloading to 15% of the peak load at a rate equal to half that used during loading. At the end of the unloading cycle, the indenter was held on the surface for a period of 100 s to establish the rate of thermal drift in the machine and specimen for correction of the data, and then completely withdrawn [35].

**Figure 2:**
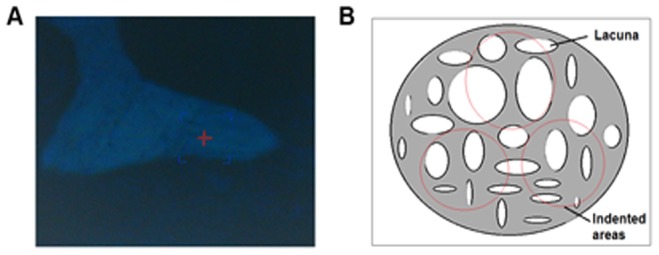
Indentation sites for nanoindentation test. Sample thickness is 2(A) Actual indented sites marked by red cross under optical microscopy; (B) Sketch map of indented areas marked by red circle.



All indents were conducted at the similar site based on the optical microscopy observation to eliminate any local effects (Fig. 2A). Three indented areas (Fig. 2B) were selected for each trabecular specimen, and five indentations were made in each target area and 15 indentations were made in every sample, and the areas for each cortical specimen were similarly selected. A total of 2205 indentations were made. *E* and *H* were determined using the method of Oliver and Pharr [36]. The quantities of concern include the peak load (*P_max_*), the contact area (*A*), and the contact stiffness (*S*). The equations used to calculate hardness (*H*) and effective indentation modulus (*E_ef_*) from the measured quantities are:
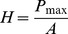
(2)and

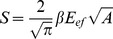
(3)The effective modulus (*E_ef_*) for the indentor-specimen combination can be derived from
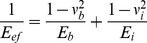
(4)


Where *ν_b_* = 0.3 and *E_b_* are Poisson’s ratio and elastic modulus for the bone material, respectively; *ν_i_ = *0.07 and *E_i_* = 1140 GPa are the same quantities for the indenter, and the factor *β* = 1.034 is a constant for the Berkovich indenter. The basic assumption involved in this method is that the sample behaves purely elastically during unloading. Any indentation close to the mounting PMMA was removed to minimize the effects of embedding on the measurements.

### Quantitative Chemical Analyses

Trabecular bones in each group were powdered to micron-sized particles using an electric grinder (Bosch Mkm 6000), and seven samples were prepared. Inductively coupled plasma optical emission spectroscopy (ICP-OES; 1.15 kW, 27 MHz; IRIS Intrepid, Thermo Electron Corporation, USA) was used for quantitative chemical analyses [37]. The user-friendly Quick Quant scan-based procedure was used to compare the intensities for measured elements in the samples with the intensities measured for standards with known concentrations. Calibration curves were calculated, and the concentrations of the measured elements in the samples were determined.

### Statistical Analysis

Differences of all groups in the macro- and micromechanical and morphological properties of femurs were analyzed using Kruskal-Wallis H test of K independent sample nonparametric test. After that, Nemenyi test of two independent samples was used to determine differences of all mechanical and morphological parameters between every two groups. Data analysis was performed with SPSS 16.0 software, and the significance level was 0.05.

## Results

### Microarchitecture of Proximal Femurs Evaluated by Micro-CT Scanning

**Figure 3:**
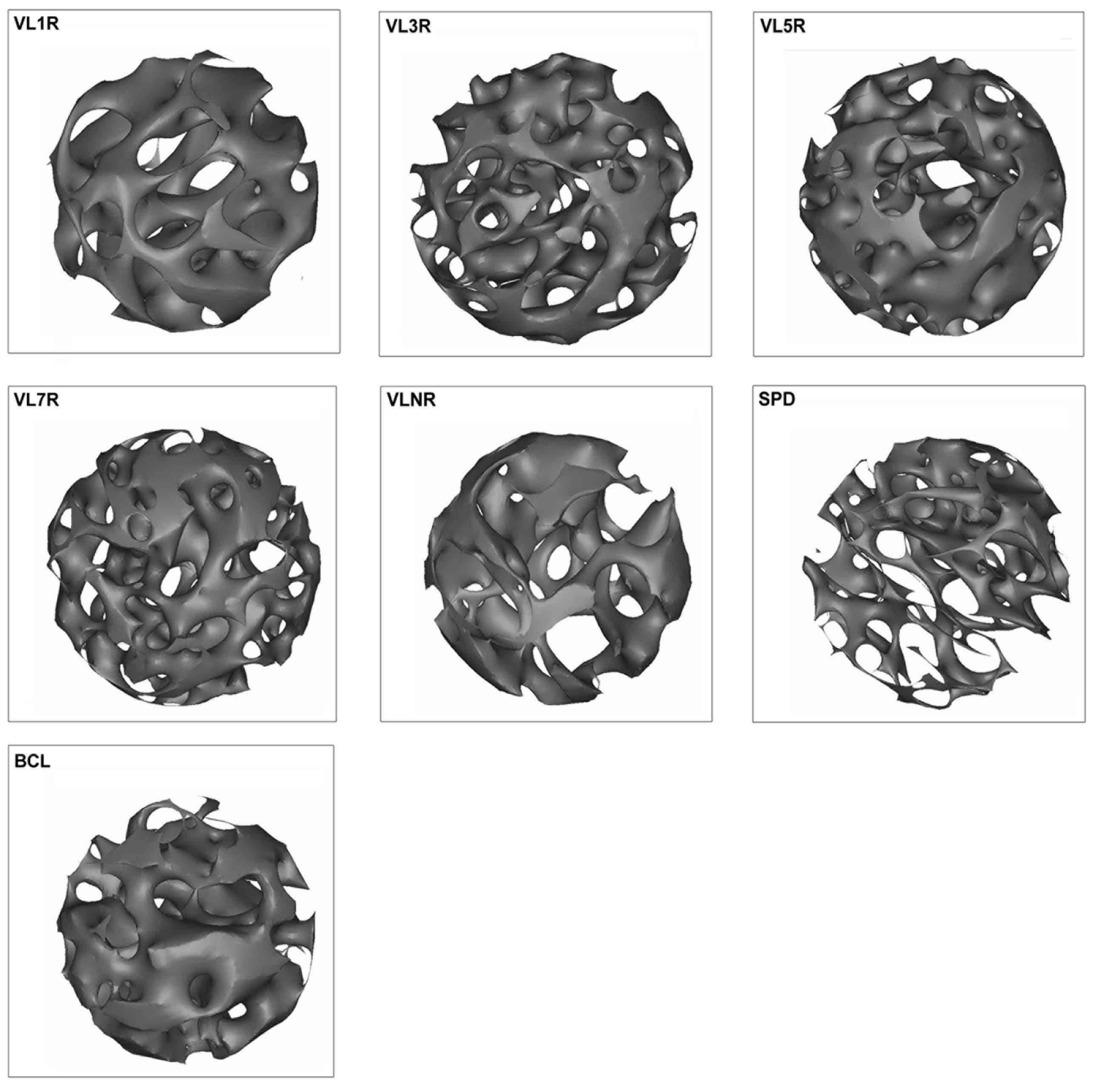
3D reconstruction of micro-CT images of trabecular bone in femoral heads. VL1R: vibrational loading for 1 d followed by 1 d rest group; VL3R: vibrational loading for 3 d followed by 3 d rest group; VL5R: vibrational loading for 5 d followed by 5 d rest group; VL7R: vibrational loading for 7 d followed by 7 d rest group; VLNR: vibrational loading for no rest day or vibrational loading every day; SPD: tail suspension group; BCL: baseline control group.

The region of interest (ROI) in the micro-CT image was selected one by one manually. The trabecular bones of femoral heads were included in the ROI as much as possible, and the 3D microarchitecture parameters calculated from CTAn are shown in Table 1. The three-dimensional (3D) reconstruction of micro-CT images of the trabecular bone in the femoral head is shown in Fig. 3. Compared with SPD, the trabeculae in the BCL are much denser, thicker and inseparable, whereas VL7R created a major improvement of trabecular microarchitecture compared with SPD, in which trabecular density, thickness and continuity were improved after eight weeks mechanical interventions. Table 1 shows variances in microarchitecture parameters of the different groups. BMD and BV/TV in all experimental groups were statistically higher than those in SPD (P<0.05), and VL7R exhibited significantly higher values than VLNR (P<0.05) and other vibrational loading groups (P<0.05), and there were statistical difference between VL1R, VL3R and VL5R (P<0.05). Furthermore, all these mechanical interventions with rest days displayed greater Tb.Th than daily loading (P<0.05), whereas VL7R showed the most obvious increase (P = 0.006). Dramatic increases in Tb.N of the femoral head were observed in all mechanical interventions, and the maximum was observed in VL7R than that in VLNR (P = 0.001). A statistically lower Tb.Sp was detected in the mechanical interventions with rest days than that in daily loading (P<0.05). There were improvements in all above microarchitecture parameters in VL1R, VL3R and VL5R than VLNR (P<0.05), but the statistical results between VL1R, VL3R and VL5R were not the same.

**Table 1:** Microarchitecture parameters evaluated by micro-CT scanning.

	VL1R	VL3R	VL5R	VL7R	VLNR	SPD	BCL
BMD (g/cm^3^)	0.70±0.02^a^^,^ b ^,^ c ^,^ d ^,^ e ^,^ f	0.74±0.03^a^^,^ b ^,^ c ^,^ e	0.65±0.01^a^^,^ b ^,^ c ^,^ d	0.79±0.11^a^^,^ b ^,^ c	0.61±0.10^a^^,^ b	0.58±0.07^b^^,^ c	0.91±0.14^a^^,^ c
BV/TV (%)	72.06±1.26^a^^,^ b ^,^ c ^,^ d ^,^ e ^,^ f	77.41±1.47^a^^,^ b ^,^ c ^,^ d ^,^ e	83.87±2.35^a^^,^ b ^,^ c	87.04±2.84^a^^,^ b ^,^ c	62.87±1.86^a^^,^ b	50.43±2.31^b^^,^ c	89.62±2.17^a^^,^ c
Tb.Th (mm)	0.21±0.01^a^^,^ b ^,^ c ^,^ e	0.22±0.03^a^^,^ b ^,^ c ^,^ e	0.19±0.01^a^^,^ b ^,^ c ^,^ d	0.22±0.01^a^^,^ b ^,^ c	0.15±0.11^a^^,^ b	0.13±0.00^b^^,^ c	0.26±0.05^a^^,^ c
Tb.N (mm^−1^)	3.22±0.14^a^^,^ b ^,^ c ^,^ d ^,^ f	4.18±0.82^a^^,^ b ^,^ c ^,^ d ^,^ e	3.48±0.24^a^^,^ b ^,^ c ^,^ d	4.92±0.41^a^^,^ b ^,^ c	3.27±0.08^a^^,^ b	2.20±0.11^b^^,^ c	5.43±0.21^a^^,^ c
Tb.Sp (mm)	0.15±0.01^a^^,^ b ^,^ c ^,^ d ^,^ f	0.12±0.02^a^^,^ b ^,^ c ^,^ e	0.14±0.01^a^^,^ b ^,^ c ^,^ d	0.11±0.01^a^^,^ b ^,^ c	0.18±0.03^a^^,^ b	0.20±0.02^b^^,^ c	0.09±0.04^a^^,^ c

n = 7 values per group. Values are shown as the median±SE. BMD - bone mineral density; BV/TV - bone volume fraction; Tb.Th - trabecular thickness; Tb.N - trabecular number; Tb.Sp - trabecular separation.

a Statistically different from SPD (P<0.05).

b Statistically different from BCL (P<0.05).

c Statistically different from VLNR (P<0.05).

d Statistically different from VL7R (P<0.05).

e Statistically different from VL5R (P<0.05).

f Statistically different from VL3R (P<0.05).

### Failure Load, Elastic Modulus, and Energy Absorption Measured by Three-point Bending Test

**Figure 4:**
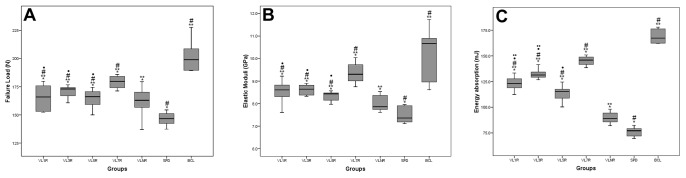
Macro-biomechanical parameters measured by three-point bending test. (A) Failure load; (B) Elastic modulus; (C) Energy absorption. *Statistically different from BCL (P<0.05). **Statistically different from SPD (P<0.05). ^#^Statistically different from VLNR (P<0.05). ^·^Statistically different from VL7R (P<0.05). ^··^Statistically different from VL5R (P<0.05).

The macro-biomechanical parameters measured by three-point bending test are shown in Fig. 4. BCL had the maximum failure load, elastic modulus, and energy absorption (P<0.05), whereas SPD had the minimum values (P<0.05). For failure load, a significant enhancement was observed in all experimental groups than that in SPD, and a significant increase was exclusively observed in VL7R than that in VLNR (P = 0.006) and other vibrational loading groups (P<0.05) (Fig. 4A). Dramatic increases in elastic modulus and energy absorption were observed in all experimental groups than those in SPD. Among all the vibration groups with rest days, elastic modulus and energy absorption were significantly higher in VL7R than those in VLNR (P<0.05) and other vibrational loading groups (P<0.05) (Figs. 4B and 4C), and there were no statistical difference between VL1R, VL3R and VL5R (Fig. 4).

### Nanostructure of Bone Material Tested by AMF

**Figure 5:**
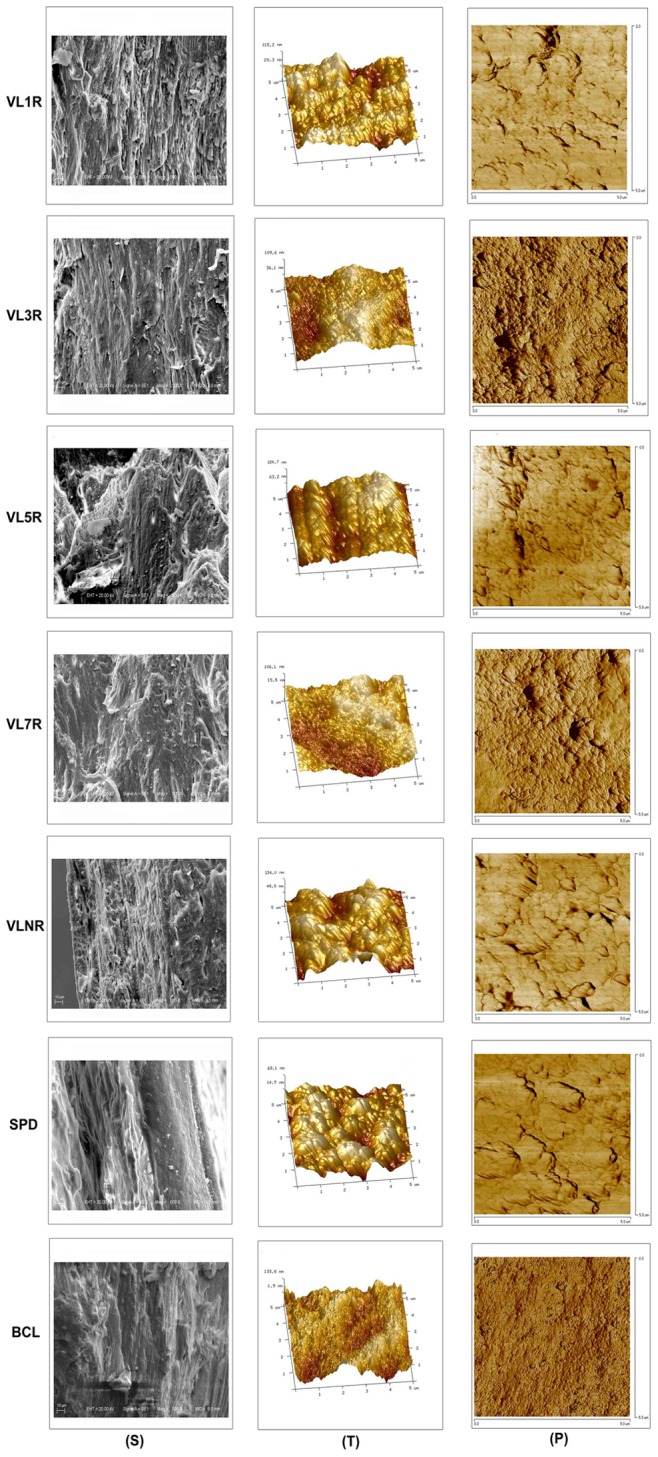
Scanning electron microscopy (SEM) images (Mag = 500X), AFM topographic images (5 μm×5 μm) and phase images (5 μm×5 μm) of the trabecular bone in femoral head. Sample thickness is 2 mm. Column (S): scanning electron microscopy; Column (T): AFM topographic images; Column (P): AFM phase images. Row VL1R: vibrational loading for 1 d followed by 1 d rest group; Row VL3R: vibrational loading for 3 d followed by 3 d rest group; Row VL5R: vibrational loading for 5 d followed by 5 d rest group; Row VL7R: vibrational loading for 7 d followed by 7 d rest group; Row VLNR: vibrational loading for no rest day or vibrational loading every day; Row SPD: tail suspension group; Row BCL: baseline control group.

Typical AFM topographic images and phase images of the nanostructure of trabecular bone material in the femoral head are shown in Fig. 5. Figs. 5-(T) show topographic images of VL1R, VL3R, VL5R, VL7R, VLNR, SPD and BCL. Scanning electron microscopy (SEM) (Figs. 5-(S)) revealed that bone minerals are fused together and form a sheet-like structure in a coherent manner [38]. The AFM phase Figs. 5-(P) obtained in our study show that the observed nanostructure of trabecular bone material exhibited a continuous phase, which is consistent with the SEM; nevertheless, the granular organization of the phase was evident in our sample (Figs. 5-(P)). The grain sizes of trabecular bone and cortical bone are listed in Table 2. Significant increases in grain sizes were observed in Figs. 5-(T)-SPD than those in Figs. 5-(T)-BCL, which showed a minimum grain size and a significantly narrower range (77 nm to 113 nm, Table 2). By contrast, the maximum values were detected in Figs. 5-(T)-SPD (P<0.05). Grain size was significantly smaller in Figs. 5-(T)-VL7R than that in Figs. 5-(T)-SPD among all the vibration groups with rest days (P<0.05). Grain size of Figs. 5-(T)-VL7R, Figs. 5-(T)-VL7R and Figs. 5-(T)-VL7R were improved than Figs. 5-(T)-VLNR (P<0.05), and statistical difference was detected between VL1R, VL3R and VL5R (P<0.05). The nanostructure of cortical bone material was similar to that of trabecular bone, and the size of grains differed among all experimental groups. Moreover, Figs. 5-(T)-SPD showed an unexpectedly low mean saturation roughness of the trabecula (approximately 70 nm, P<0.05), which showed that larger grains were flattened. By contrast, the mean saturation roughness values in Figs. 5-(T)-BCL and Figs. 5-(T)-VL7R were significantly higher (approximately 110 nm to 130 nm, P<0.05).

**Table 2:** Grain size of trabecular bone and cortical bone.

	VL1R	VL3R	VL5R	VL7R	VLNR	SPD	BCL
Tb.(nm)	294±43^a^^,^ b ^,^ c ^,^ d ^,^ e ^,^ f	347±31^a^^,^ b ^,^ c ^,^ d ^,^ e	415±39^a^^,^ b ^,^ c ^,^ d	220±21^a^^,^ b ^,^ c	493±38^a^^,^ b	684±57^b^^,^ c	95±18^a^^,^ c
Ct.(nm)	316±23^a^^,^ b ^,^ c ^,^ d ^,^ e ^,^ f	359±34^a^^,^ b ^,^ c ^,^ d ^,^ e	413±37^a^^,^ b ^,^ c ^,^ d	197±16^a^^,^ b ^,^ c	521±43^a^^,^ b	659±49^b^^,^ c	110±13^a^^,^ c

n = 7 values per group. Sample thickness is 2 mm. Values are shown as the median±SE. Tb. - trabecular bone; Ct. - cortical bone.

a Statistically different from SPD (P<0.05).

b Statistically different from BCL (P<0.05).

c Statistically different from VLNR (P<0.05).

d Statistically different from VL7R (P<0.05).

e Statistically different from VL5R (P<0.05).

f Statistically different from VL3R (P<0.05).

### Bone Growth Measured by Fluorescence Test

**Figure 6:**
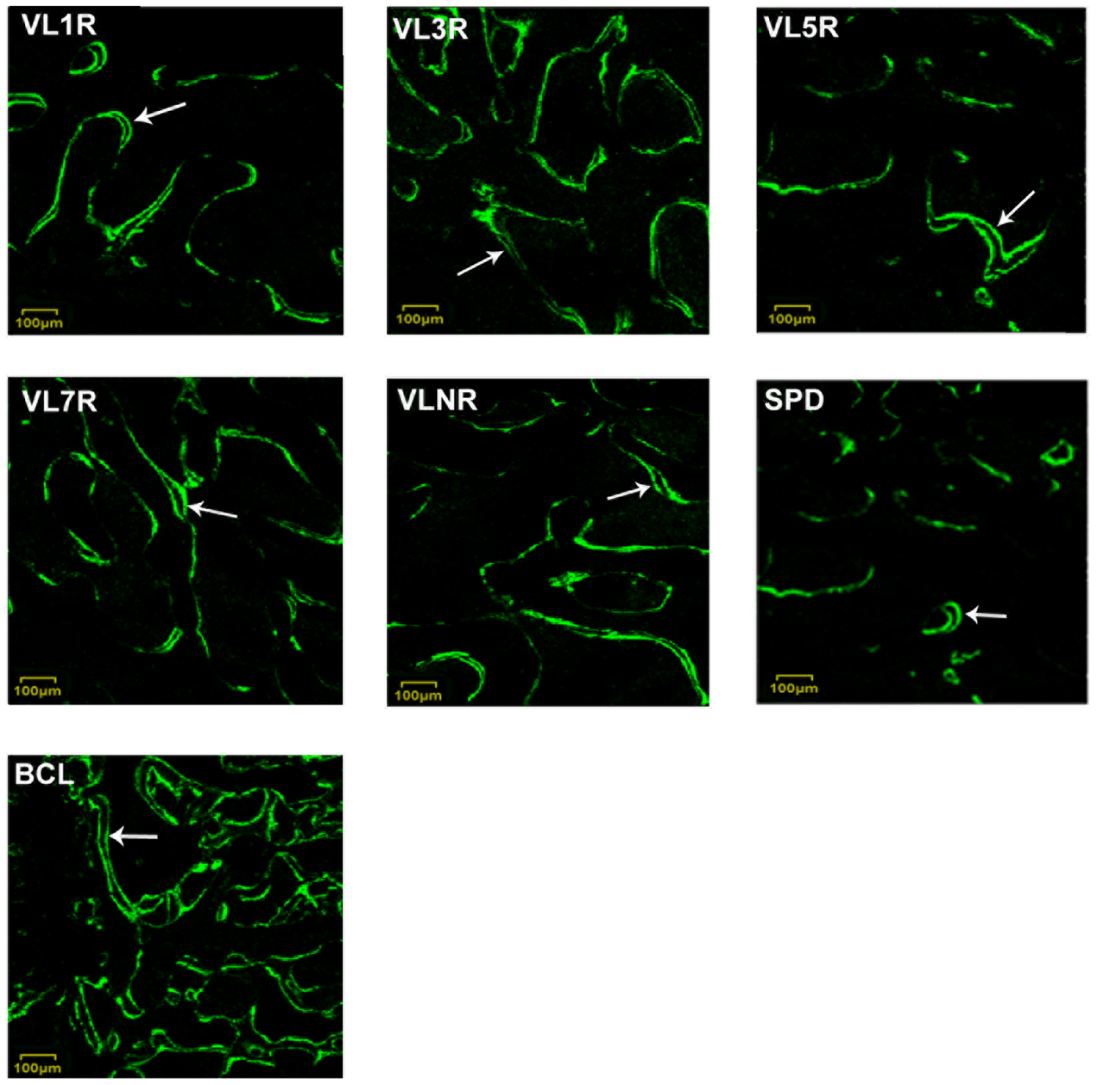
Fluorescence graphs of cancellous bones in proximal femurs. Arrows show the areas where the mineral apposition rate and bone formation rate were measured. VL1R: Vibrational loading for 1 d followed by 1 d rest group; VL3R: Vibrational loading for 3 d followed by 3 d rest group; VL5R: Vibrational loading for 5 d followed by 5 d rest group; VL7R: Vibrational loading for 7 d followed by 7 d rest group; VLNR: Vibrational loading for no rest day or vibrational loading every day; SPD: Tail suspension group; BCL: Baseline control group.

**Figure 7:**
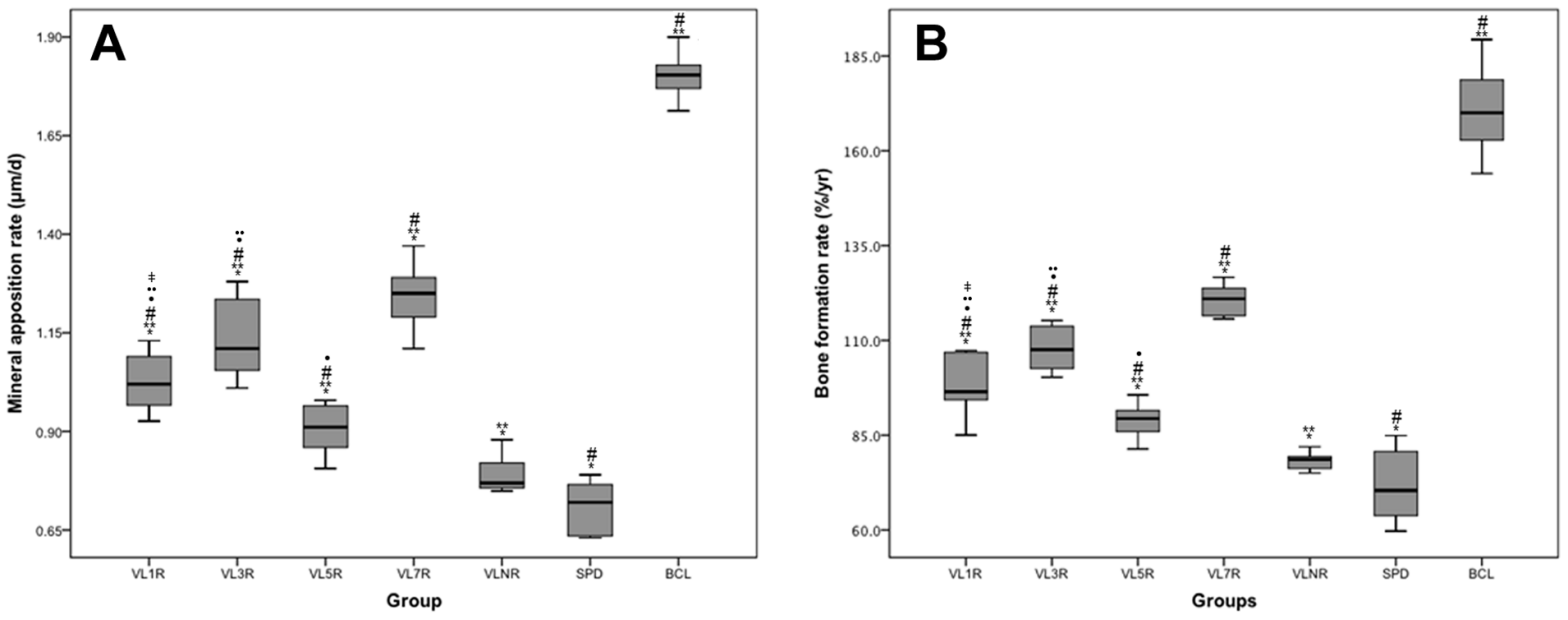
Mineral apposition rate and bone formation rate. *Statistically different from BCL (P<0.05). **Statistically different from SPD (P<0.05). ^#^Statistically different from VLNR (P<0.05). ^·^Statistically different from VL7R (P<0.05). ^··^Statistically different from VL5R (P<0.05). ^‡^Statistically different from VL3R (P<0.05).

Fluorescence marker lines were clearly observed in the fluorescence graph in Fig. 6. The MAR and BFR/Tb.Ar are shown in Fig. 7. Maximum values were detected in BCL (P<0.05), whereas minimum values were detected in SPD (P<0.05). A significant increase was exclusively found in VL7R than that in VLNR (P = 0.001) and other vibration groups with rest days (P<0.05). The MAR and BFR/Tb.Ar were increased in VL1R, VL3R and VL5R than VLNR (P<0.05), and there were statistical difference between VL1R, VL3R and VL5R (P<0.05).

### E and H of Bone Material Evaluated by Nanoindentation Test

A summary of *E*, *H*, and *E/H* ratio of the trabecular and cortical bone sites as determined by nanoindentation is shown in Table 3. All data were obtained at an indentation depth of approximately 1000 nm [35]. *E*, *H*, and *E/H* ratio of longitudinal cortical bone (Ct.L) were greater than those of longitudinal trabecular bone (Tb.L), followed by transverse trabecular bone (Tb.T). No statistical difference was found in *E*, *H*, and *E/H* ratio for all groups (P_Tb.T_ = 0.845, P_Tb.L_ = 0.172, and P_Ct.L_ = 0.100 for *E*; P_Tb.T_ = 0.707, P_Tb.L_ = 0.414, and P_Ct.L_ = 0.179 for *H*; P_Tb.T_ = 0.898, P_Tb.L_ = 0.540, and P_Ct.L_ = 0.231 for *E/H* ratio).

**Table 3:** *E*, *H*, and *E/H* ratio of the trabecular and cortical bone sites as determined by nanoindentation.

		VL1R	VL3R	VL5R	VL7R	VLNR	SPD	BCL
Tb.T	*E* (GPa)	14.90±1.02	15.00±1.11	15.00±1.23	15.00±0.89	14.90±0.74	14.90±1.35	15.00±1.41
	*H* (GPa)	0.20±0.01	0.20±0.01	0.19±0.03	0.20±0.02	0.19±0.03	0.20±0.02	0.20±0.01
	*E/H*	74.50±3.41	75.00±4.29	77.90±2.79	74.00±2.76	77.90±4.73	75.00±1.96	75.00±2.47
Tb.L	*E* (GPa)	23.72±2.11	23.50±2.64	23.58±1.89	23.16±2.13	23.20±2.29	23.65±3.01	23.15±1.74
	*H* (GPa)	1.24±0.03	1.23±0.11	1.20±0.10	1.13±0.09	1.15±0.03	1.19±0.10	1.21±0.06
	*E/H*	19.37±2.12	19.15±1.71	19.72±1.22	20.39±1.59	20.05±2.85	19.87±1.97	19.40±2.18
Ct.L	*E* (GPa)	30.20±2.94	30.20±3.15	30.20±2.76	30.40±2.18	30.60±3.31	30.10±2.87	30.80±2.09
	*H* (GPa)	1.49±0.12	1.49±0.09	1.49±0.04	1.49±0.01	1.49±0.01	1.49±0.04	1.50±0.10
	*E/H*	20.34±1.86	20.20±1.43	20.34±0.98	20.54±1.03	20.54±2.10	20.20±1.74	20.53±1.35

n = 7 values per group. Sample thickness is 2 mm. Values are shown as the median±SE. Tb.T - transverse trabecular bone; Tb.L - longitudinal trabecular bone; Ct.L - longitudinal cortical bone; *E* - elastic modulus; *H* - hardness; *E/H* - ratio of elastic modulus and hardness.

### Bone Composition Evaluated by Quantitative Chemical Analyses

Results of quantitative chemical analyses (Table 4) show unchanged levels of calcium and phosphorus in all groups (P = 0.983), and no significant difference was found in the Ca/P ratio (P = 0.991).

**Table 4:** Chemical composition of trabecular bone in femoral head (ICP-OES).

	VL1R	VL3R	VL5R	VL7R	VLNR	SPD	BCL
Ca [%]	24.34±2.51	24.92±1.91	24.59±2.55	24.72±3.02	24.82±2.37	24.51±2.62	24.59±2.29
P [%]	11.32±0.42	11.40±0.23	11.44±0.21	11.56±0.32	11.43±0.24	11.39±0.40	11.38±0.25
Ca/P ratio	2.15±0.19	2.19±0.21	2.15±0.23	2.14±0.17	2.18±0.21	2.15±0.19	2.16±0.23
Mg [%]	0.46±0.04	0.47±0.02	0.50±0.01	0.50±0.01	0.50±0.03	0.46±0.02	0.48±0.01
Sr [%]	0.0070±0.00	0.0069±0.00	0.0066±0.00	0.0069±0.00	0.0069±0.00	0.0067±0.00	0.0070±0.00
Fe [%]	0.015±0.00	0.016±0.00	0.013±0.00	0.015±0.00	0.012±0.00	0.013±0.00	0.023±0.00
Ba [%]	<0.005±0.00	<0.005±0.00	<0.005±0.00	<0.005±0.00	<0.005±0.00	<0.005±0.00	<0.005±0.00
K [%]	0.093±0.01	0.092±0.01	0.11±0.02	0.10±0.01	0.10±0.01	0.087±0.01	0.098±0.01
Na [%]	0.61±0.03	0.67±0.02	0.78±0.01	0.70±0.01	0.72±0.02	0.61±0.01	0.70±0.02

n = 7 values per group. Values are shown as the median±SE. ICP-OES - inductively coupled plasma optical emission spectroscopy.

## Discussion

BMD decreased when hindlimbs were suspended and high-frequency, and low-magnitude mechanical stimuli could improve the biomechanical properties and microarchitecture in various sites, including cancellous and shaft cortical bones [39]–[42]. Whole-body vibration mitigated the reduction of bone strength in long-term hindlimb unloading rats and the complexity of trabecular bone could be preserved [43]–[45]. Low-magnitude, high-frequency mechanical stimuli may provide a non-pharmacologic treatment for bone loss. But bone cells lose mechanical sensitivity soon after stimulation, and a rest interval between each low-magnitude load cycle may create a potent anabolic stimulus [11]–[13]. Therefore, high-frequency, low-magnitude whole body vibration with rest days was put forward. However, the most effective number of rest days for treating bone loss was still unknown. Based on the 5 working days, VL5R was established in this study, and two smaller groups, i.e. VL1R and VL3R and one bigger group, i.e. VL7R were created. Groups of vibrational loading every day (VLNR), tail suspension (SPD), and baseline control (BCL) were also included for comparison to investigate multi-level mechanical properties, morphology, and chemical composition of bone to explore the effects of high-frequency, low-magnitude whole body vibration with different rest days.

In this study, macromechanical properties (i.e., failure load, elastic modulus, and energy absorption) increased in all experimental groups than those in SPD, and VL7R was significantly higher than VLNR (P<0.05) and other vibration groups with rest days (P<0.05). Similar results were detected in microarchitecture parameters, including increased BV/TV, BMD, Tb.Th, Tb.N, and lower Tb.Sp, of VL7R than those of VLNR (P<0.05) and other vibration groups with rest days (P<0.05). A 3D reconstruction of micro-CT images (Fig. 3) and fluorescence graphs (Fig. 7) showed that the trabeculae of BCL were significantly denser and thicker than SPD or vibrational loading groups, and bone growth in BCL was significantly greater than that in other groups. By contrast, significant trabecular deterioration was observed in rodents after tail suspension without mechanical intervention. Stimuli with rest days resulted in a major improvement of trabecular microarchitecture than SPD such that trabecular density, thickness, and continuity improved and bone growth rate was higher, and VL7R was more effective than VL1R, VL3R or VL5R. Whole-body vibration mitigated the reduction of bone strength in long-term hindlimb unloading rats, and mechanical stimulation in the form of whole-body vibration limited reduction of bone density when it was applied during the unloading [43], [45], which was similar to the results of this study. However, our study showed that the vibrational loading for 7 d followed by 7 d rest appeared to be the optimal loading strategy for the bone loss caused by mechanical disuse, since significant differences were found in all above parameters.

The AFM phase images obtained in this study show that the observed nanostructure of the bone material exhibited a continuous phase and evident granular structure, which was consistent with the observation of Milovanovic et al. [25]. Compared with BCL, the grain sizes in all experimental groups were larger. In addition to crystal growth, two additional processes can increase mineral size, namely, crystal secondary nucleation leading to crystal proliferation and aggregation of preformed crystals [46]. Increased grain size in SPD cannot be explained by the increase in amount of minerals because our quantitative chemical analyses revealed unchanged calcium and phosphorus levels, as well as Ca/P ratio, in all experimental groups. Our findings indicate that in SPD, the existing mineral was reorganized by aggregation to larger grains, similar to the suggestion for turkey-leg tendon maturation [47], [48]. Lower roughness in SPD, despite its larger grains, also supported the reorganization hypothesis, in which fused grains formed a larger flattened structure. Small mineral grain size of vibrational groups in this study was attributed to new bone formed in the remodeling process [49], and in all vibration groups with rest days, mineral grain size of VL7R was significantly smaller than VLNR (P<0.05) and other vibration groups with rest days (P<0.05), which showed that VL7R was more efficacious in improving bone loss and promoting bone formation. By contrast, large grains were possibly located in sites that have not undergone the bone remodeling process for a considerable amount of time. However, further experimental studies are needed for direct investigation of the mechanisms of grain enlargement. Nanoindentation test was used in this study to investigate the material properties of trabecular and cortical bones. Nanoindentation, which is a different method from conventional microhardness techniques, measures relatively smaller areas of bone material and provides estimates for both *E* and *H*. In this study, *E* and *H* of trabeculae and cortical bones were measured, and the average *E* and *H* of trabecular bone were considerably smaller than those of cortical bone (P<0.05). The *E/H* ratio, which represents the overall behavior of bone during the indentation process with respect to fracture toughness, was useful to describe material deformation during indentation and determine the brittleness of a material (ductile materials with higher *E/H* value) [50]. However, no significant difference in bone material properties was observed among all groups (P>0.05). Thus, the effect of disuse on the collagen bone matrix should be investigated. Other studies found that *E* and *H* of lamellae are unrelated to age, gender, and body mass index, and reductions in the mechanical integrity of whole bone must be caused by other factors, such as changes in tissue mass and organization [51], [52]. Many of these changes are possibly caused by structural and histological features rather than alterations in the bone matrix itself, and these changes warrant further investigation [53], [54].

Some investigations suggested that high-frequency and low-magnitude mechanical signal may suppress adipose, non-esterified free fatty acid, and triglyceride contents in the liver or reduce a downstream challenge to liver morphology and function in rodents fed with a high-fat diet [55], [56]. This mechanical efficacy to inhibit adipogenesis may increase with additional loading bouts if a refractory period is incorporated [57]. In this study, weights of animals sustained in tail suspension showed a similar ascending trend over seven weeks of vibration. However, animals loaded with mechanical stimuli appeared to lose weight in the last week (P<0.05). The variation in weight was similar to that reported by Ma et al. [14]. At the same time, hindlimb-unloading induced loss of mass in muscles, but the response of BMD to altered loading conditions did not necessarily depend on the response of muscle mass [39]. Further studies will be performed to understand the mechanism of weight loss and the response of BMD to muscle mass affected by vibration stimuli.

This study had several limitations. First, three-month-old male rats were selected to exclude the influence of hormones with respect to osteoporosis of ovariectomised rats caused by estrogen deficiency [14]. In future studies, female rats with the same age will be investigated to determine the effect of gender. Second, though three-month-old male rats have been carefully limited in this study, body mass or size of all rats was not measured daily, which could have been responsible for the differences between individuals. Likewise, the behavioral monitoring may have been able to recognize any potential differences in activity levels between animals (those that were not undergoing tail suspension), and this study did not establish a group of tail suspension with 15 min of free activities instead of vibration stimuli, but the experimental groups only had different vibration cycles, and statistical results were unaffected by free activities during the vibration period. Further studies will record body mass or size and perform the behavioral monitoring daily, which would help eliminate those from being variables that potentially cloud the results, and an experimental group with tail suspension rodents and free activities will be included to determine the influence of free activities on bone quality. Third, bilateral femurs were used because the rat femoral heads were too small to collect all necessary specimens. In relation to the investigation on Caucasian women, which showed no significant difference in BMD of bilateral femoral necks, trochanters, and hips [58], bilateral differences were ignored in this study. Fourth, the bone turnover markers P1NP and CTX-I on rat serum were not studied to quantify the difference of bone formation and bone resorption between different groups, and this will be quantified in further studies. The number of days that rats underwent vibrational loading and rest is smaller than seven. However, vibrational loading for X day followed by X day rest (VLXR, X = 1, 3, 5, 7) were studied in contrast with vibrational loading every day (VLNR), and this study showed that high-frequency, low-magnitude whole body vibration with rest days was efficacious in improving bone quality than daily loading. In future studies, the days of vibrational loading and rest will be diversified. Fifth, the samples were dehydrated before the experiment, which were different from fresh samples. Because the quantity of samples was limited, and the same sample was used for a variety of experimental study. Thus, considering the different experimental needs, all the samples were dehydrated in the same condition, which would not affect the statistical significance. The mechanical experiments on fresh samples will be carried out in the later study to make it closer to the reality.

This study showed that bone mineral reorganization and spatial arrangement were modified by high-frequency, low-magnitude whole body vibration with different rest days, which resulted in the recombination of mineral grains into different sizes and changes in mineral apposition rate and bone formation rate. The microarchitecture of bone was affected, which resulted in statistical differences in macromechanical properties. However, the mechanical properties of the bone material were not altered. This study investigated the influence of high-frequency, low-magnitude whole body vibration on bone loss caused by mechanical disuse in multiple levels, and showed that high-frequency, low-magnitude whole body vibration with 7 d rests was more efficacious in improving macro-biomechanical properties and microarchitecture than daily loading, which provided a theoretical basis for improving bone quality using this mechanical intervention in clinics.
